# Dynamic functional connectivity patterns predict early antidepressant treatment response in drug-naïve, first-episode adolescent MDD

**DOI:** 10.3389/fnins.2025.1487754

**Published:** 2025-02-03

**Authors:** Maojia Ran, Hang Zhang, Meijiang Jin, Yuanmei Tao, Hanmei Xu, Shoukang Zou, Zhujun Wang, Fang Deng, Lijuan Huang, Hong Zhang, Xiaowei Tang, Yanping Wang, Xia Fu, Li Yin

**Affiliations:** ^1^Department of Psychiatry, West China Hospital of Sichuan University, Chengdu, Sichuan, China; ^2^Frontier Science Center for Disease-related Molecular Networks, Chengdu, Sichuan, China; ^3^Sichuan Clinical Medical Research Center for Mental Disorders, Chengdu, Sichuan, China

**Keywords:** adolescent, MDD, Dfc, antidepressant, treatment response, MRI

## Abstract

**Objective:**

Adolescents with major depressive disorder (MDD) exhibit abnormal dynamic functional connectivity (dFC) patterns, but it remains unclear whether these aberrant dFC patterns are linked to antidepressant treatment. The aim of this study is to investigate whether dFC patterns will be changed by antidepressant treatment, as well as whether baseline dFC pattern could predict treatment response in adolescent MDD patients.

**Method:**

We included 35 drug-naïve, first-episode MDD adolescents (age 14.40 ± 1.24; 8 males and 27 females) and 24 healthy controls (HCs, age 14.21 ± 1.41; 11 males and 13 females). All MDD adolescents received 6 weeks of antidepressant treatment. Resting state and T1 MRI data were collected in MDD adolescents before and after treatment and in HCs. Independent component analysis (ICA) was used to compare the different dFC pattern between MDD adolescents and HCs at baseline, as well as which between before and after treatment in MDD adolescents. Finally, Pearson correlation and multivariate linear regression analyses were used to explore the associations between dFC pattern and changed score of BDI in MDD adolescents.

**Results:**

The mean dFC value between right inferior frontal gyrus (IFG) and bilateral insular cortex (IC; right, *r* = −0.461, *p*-FDR = 0.012; left, *r* = −0.518, *p*-FDR = 0.007) at baseline were negatively correlated with BDI score reduction. The mean dFC value between left frontal pole (FP) and right superior parietal lobule (SPL) after treatment was positively correlated with BDI score reduction (*r* = 0.442, *p*-FDR = 0.014). And the mean dFC values between right IFG and bilateral IC (right, *β* = −1.563, *p*-FDR = 0.021; left, *β* = −1.868, *p*-FDR = 0.012) at baseline could predict antidepressant treatment response.

**Conclusion:**

These findings demonstrate that dFC patterns between some brain areas could be a prospective factor for predicting antidepressant treatment response.

## 1 Introduction

Major depressive disorder (MDD) is a mental illness with high morbidity, mortality and recurrence rate (Demyttenaere et al., 2004; Malhi and Mann, 2018; Monroe and Harkness, 2022). It is currently the second leading cause of disability worldwide (Collaborators, 2024). Among Chinese children and adolescents, MDD has become the third most common mental illness (Li F. et al., 2022). Moreover, adolescents with MDD are prone to substance abuse and suicide (Thapar et al., 2012).

Despite widespread use of antidepressants, remission rate remains only 30 to 40% (Trivedi et al., 2006; Ioannidis, 2008; Zhou et al., 2020). In addition, slow onset of antidepressant effects also increases burdens on patients and their family (Quitkin et al., 1987). But finding predictors of treatment response has always been a challenge. Various neuroimaging markers have shown their abilities to predict antidepressant treatment response, such as gray matter volume and static functional connectivity (FC) patterns (Langenecker et al., 2007; Williams et al., 2015; Dunlop et al., 2017; Liu et al., 2017; Chin Fatt et al., 2020). While the functional connectome approach suggests that brain FC is changed over time (Calhoun et al., 2014; Marchitelli et al., 2022). The method, dynamic functional connectivity (dFC) analysis, estimates time-varying functional connectivity on a temporal scale (Cribben et al., 2012; Engel and Gerloff, 2022), and reflects more transitory patterns in blood oxygen level dependent (BOLD) signals (Allen et al., 2014; Jalilianhasanpour et al., 2021). But most of studies have ignored dFC changes associated with MDD and antidepressant treatment.

Early works have showed that MDD adolescents exhibited changed dFC strength in brain regions which involved in emotional and cognitive process (eg. prefrontal cortex and parahippocampal gyrus; Cribben et al., 2012; Kaiser et al., 2015a; Marchitelli et al., 2022). Surprisingly little is known about the relationship between dFC patterns and antidepressant treatment. Connectome-based predictive modeling demonstrated that baseline dFC within anterior cingulate cortex (ACC) predicted less improvement in cognitive flexibility after psilocybin therapy (Doss et al., 2021). Findings indicated that reward network may serve as the potential target for antidepressant treatment and the promising predictors of treatment response (Hou et al., 2018). Li et al. (2021) quantified functional stability and found that baseline functional stability in dorsal ACC and ventral ACC, calcarine sulcus, and middle occipital gyrus could be valid predictors of remission in MDD patients. But studies above had (1) small sample size of less than 30 cases, (2) short (2 weeks) or long-term (7 months) treatment response to be explored. There are also several articles about dFC patterns predicting the response of electroconvulsive therapy (ECT) or psychotherapy. For example, dFC between default mode network (DMN) and cognitive control network (CCN), could as a predictive biomarker of the outcome of ECT in depressed patients (Dini et al., 2021). MDD patients showed increased dFC variability in dorsolateral prefrontal cortex and precuneus after cognitive behavioral therapy (Zhou et al., 2021). However, these existing studies have mainly focused on a few brain regions or neural circuits, revealing a bias in dFC patterns that are particularly related to antidepressant treatment response (Voegeli et al., 2017; Kuai et al., 2024).

In this study, we sought to determine whether baseline dFC patterns could predict early antidepressant treatment response. The results have the potential to provide extensive information on the brain basis of antidepressant treatment in adolescents with MDD.

## 2 Methods

### 2.1 Participants

This study was approved by the Ethics Committee of West China Hospital, Sichuan University and registered on the Chinese Clinical Trial Registration Platform (ChiCTR2000033402). Drug-naïve, first-episode MDD adolescents and healthy controls aged 12–17 years from September 2020 to December 2021. All study subjects were from the inpatient or outpatient departments of West China Hospital of Sichuan University, and healthy controls were recruited through advertising. All participants and their guardians signed written informed consent. All adolescents with MDD were diagnosed according to the diagnostic criteria for MDD in the Diagnostic and Statistical Manual of Mental Disorders-Fourth Edition (DSM-IV). Two senior psychiatrists performed mental health examinations using the Chinese version of Affective Disorders and Schizophrenia-Present and Lifetime Version (KSADS-PL; Kaufman et al., 1997). Thirty-five adolescents with MDD (age 14.40 ± 1.24; 8 males and 27 females) and 24 healthy controls (HCs, age 14.21 ± 1.41; 11 males and 13 females) were included in this study. Inclusion criteria for both MDD patients and HCs were 12–17 years old, right-handed, have at least an elementary school education, have a normal brain structure, be able to understand the contents of the scales, IQ > 85, has not received electroconvulsive therapy, and has not taken any other medications recently. We exclude those subjects with other axis I and axis II mental illness, severe physical illnesses, history of neurologic disease or injury, previous use of illicit substances, pregnancy or breastfeeding. No HCs had psychiatric illness or had attempted suicide.

All adolescents with MDD were treated with antidepressants, either alone or in combination with antipsychotics, sedative-hypnotics or anxiolytics. Antidepressants included agomelatine (43%, 15/35, 50 mg/d), sertraline (37%, 13/35, 50–150 mg/d), escitalopram (14%, 5/35, 10–15 mg/d) and venlafaxine (6%, 2/35, 150–225 mg/d). Antipsychotics included quetiapine (14%, 5/35, 50–100 mg/d) and olanzapine (9%, 3/35, 2.5 mg/d). Sedative-hypnotics included alprazolam (26%, 9/35, 0.4 mg/d), zopiclone (9%, 3/35, 7.5 mg/d) and lorazepam (9%, 3/35, 0.5 mg/d). Anxiolytic was tandospirone (6%, 2/35, 30 mg/d). In subsequent analyses, the doses of antidepressants, antipsychotics and sedative-hypnotics were converted to equivalent doses of fluoxetine (Hayasaka et al., 2015), chlorpromazine (Andreasen et al., 2010) and diazepam (Borrelli et al., 2022), respectively.

### 2.2 Assessment

Beck Depression Inventory (BDI) was used to evaluate the depressive symptom severity of patients before and after treatment (Jackson-Koku, 2016). Patients were defined as responder group (group A, BDI score reduction ≥50%), non-responder group (group B, 25% ≤ BDI score reduction <50% and group C, BDI score reduction <25%; Reeves et al., 2012). HCs also completed the BDI at baseline.

### 2.3 MRI protocol procedure

Resting-state structural and functional MRI data were collected in MDD adolescents before and after treatment and in HCs on the same 3.0 T MRI scanner (uMR790, United-Imaging Healthcare, Shanghai, China). Participants were asked to remain calm, close their eyes, relax, and not think about anything during the scan. Foam padding and earplugs were used to minimize the effects of head movements and scanner noise. After the scan, participants were asked if they fell asleep during the scan. During the scan, images were visually inspected for structural abnormalities, head motion and artifacts. Only images without the above influence were retained for the study.

T1-weighted (T1w) images were acquired using a magnetization-prepared, rapid gradient-echo sequence. The parameters were as follows: repetition time (TR), 8.4 ms; echo time (TE), 3.8 ms; flip angle, 8°; slice thickness, 0.8 mm; total number of sagittal slices, 208; matrix size, 256 × 256; field of view (FOV), 256 × 256 mm^2^; and voxel size, 0.8 × 0.8 × 0.8 mm^3^.

Resting-state background echo-planar imaging (EPI) were obtained. The parameters were as follows: TR, 1000 ms; TE, 30 ms; flip angle, 60°; FOV, 210 × 210 mm^2^; number of slices, 65; voxel size, 2.5 × 2.5 × 2.5 mm^3^; and layer thickness, 2.5 mm.

### 2.4 Functional image preprocessing

The original dicom file was converted to nifty file using MRIcron.^1^ Then images of each subject were visually inspected again to ensure the absence of artifacts, deletions and ghosting before preprocessing. The data were preprocessed and analyzed using MATLAB R2022b (MathWorks, Natick, MA, United States), SPM12^2^ and CONN toolbox version 22b.^3^ The steps for preprocessing the structural MRI data included translation to the (0, 0, 0) coordinates, segmentation and normalization (using gray/white/cerebrospinal fluid segmentation and MNI normalization). T1W image was used for segmentation because it has higher spatial resolution. Since we are ultimately interested in functional image data, we then had to resample these tissue maps onto the functional data. Functional pre-processing involved the following steps: realignment and unwarp (for motion and field map correction), translation of the image center (to the origin 0, 0, 0), slice-timing correction, outlier scan detection and scrubbing (using ART: artifact removal toolbox), spatial normalization to an MNI template (functional target resolution 2 mm) and functional smoothing (full width half maximum of 8 mm). Default preprocessing parameters were used to define and reduce possible confounding due to head movement and blood oxygen level-dependent signals in cerebrospinal fluid and white matter. For noise reduction due to physiological effects (such as respiration and pulsation) and drift caused by scanner noise, the signals for white matter and cerebrospinal fluid were regressed from the functional data, and a bandpass filter of 0.008–0.09 Hz was applied by default (Hallquist et al., 2013). After preprocessing, quality control was performed for each subject.

### 2.5 Dynamic independent component analysis

The ICA can separate signals from noise and increase the sensitivity of detecting inter-individual differences (Monakhova and Rutledge, 2020). Dyn-ICA matrices represent a measure of different modulatory circuits expression and rate of connectivity change between each pair of regions of interest (ROIs), characterized by the strength and sign of connectivity changes covarying with a given component/circuit timeseries. In this research, dynamic connectivity analysis was performed to explore cross-time functional modulation in the matrix of connectivity between ROIs (Calhoun et al., 2014). The dyn-ICA was conducted using the CONN toolbox and performed on the connectivity time-series, calculated the connectivity strength between any pair of ROIs at any given time point. The computation involved ICA decomposition of the ROI-ROI dynamic connectivity timeseries into 20 components with 30 smoothing kernels (selected by default in CONN). CONN’s dyn-ICA implementation follows Calhoun’s group-ICA methodology (Calhoun et al., 2001), with optional subject-level dimensionality reduction, concatenation across subjects, iterative dual regression on group-level data, a fastICA algorithm for group-level independent component definition (with a hyperbolic tangent contrast function), and subject-level back-projection (back-projection of the group-level connectivity matrices into a series of subject-specific connectivity components). Then, T tests (including independent and paired T tests) were used to compare different dFC patterns between MDD and HCs, as well as between before and after treatment. Threshold-free cluster enhancement was used for multiple comparison correction (Smith and Nichols, 2009). These ROIs were defined from the brain atlas file in the CONN toolbox and included 132 brain regions derived from the Harvard-Oxford Cortex Atlas (provided by the Harvard Center of Morphometric Analysis, Cambridge, Massachusetts) and cerebellum parcellations.

### 2.6 Statistical analysis

We extracted the dFC values between each two brain areas at different time points and calculated the average value of the entire scan time. Pearson correlation was used to investigate the association between mean dFC values (before and after treatment) and BDI score (BDI score reduction, BDI score before and after treatment) in MDD patients. Multivariate linear regression analyses were carried out to determine whether dFC patterns at baseline can predict antidepressant treatment response. Covariates included age, sex, baseline BDI score and medication dosage (including dose of antidepressant, antipsychotics, sedative-hypnotics and anxiolytics) of patients. To validate the prediction analyses, we conducted repeated 3-fold cross-validation (Ash and Hughes-Oliver, 2018) and permutation test. Cross-validation is virtually unbiased, but is known to be variable when applied to small samples (Li et al., 2022). To reduce its variability, the whole process was repeated 10 times. While permutation test was most used statistical analysis for small sample studies (Liu, 2024). We also conducted sensitivity and specificity analyses for each dFC pattern identified as a predictor to identify neuroimaging biomarkers of differential treatment response. An adjusted *p* value <0.05 [False Discovery Rate (FDR) correction] was used. All statistical analyses were performed using R 4.3.3.^4^

## 3 Results

### 3.1 Demographic characteristics

Demographic and clinical characteristics of the participants are listed in Table 1. There were no statistical differences in sex distribution ( $x^{2}$ = 2.471, *p* = 0.116) or age distribution (*t* = −0.551, *p* = 0.584) between HC and MDD group. In MDD patients, the BDI scores decreased significantly after treatment (*t* = 4.226, *p* < 0.001). 14% of patients achieved treatment response (Group A, 5/35), while 86% of patients failed to achieve response (Group B, 29%, 10/35 and Group C, 57%, 20/35).

**Table 1:** Sociodemographic and clinical characteristics of the participants.

	HCs (*n* = 24)	MDD adolescents (*n* = 35)	*x*^2^ / *t*	*p*	Effect size	MDD adolescents		*x*^2^ / *t*	*p*	Effect size
						Responders (*n* = 5)	Non-responders (*n* = 30)			
	N (%)	N (%)	*x* ^2^	*p*	Hedges’ g	N (%)	N (%)	*x* ^2^	*p*	Hedges’ g
Sex			2.471	0.116	0.413			0.972	0.568	0.330
Male	11 (46)	8 (23)				2 (40)	6 (20)			
Female	13 (54)	27 (77)				3 (60)	24 (80)			
Antidepressants			NA	NA	NA			3.390	0.268	0.640
Agomelatine	NA	15 (43)				3 (60)	12 (40)			
Sertraline	NA	13 (37)				1 (20)	12 (40)			
Escitalopram	NA	5 (14)				0	5 (17)			
Venlafaxine	NA	2 (6)				1 (20)	1 (3)			
Antipsychotics			NA	NA	NA			0.972	0.568	0.330
Yes	NA	8 (23)				2 (40)	6 (20)			
No	NA	27 (77)				3 (60)	24 (80)			
Sedative-hypnotics			NA	NA	NA			0.019	0.889	0.046
Yes	NA	15 (43)				2 (40)	13 (43)			
No	NA	20 (57)				3 (60)	17 (57)			
Anxiolytics			NA	NA	NA			0.354	0.552	0.198
Yes	NA	2 (6)				0	2 (7)			
No	NA	33 (94)				5 (100)	28 (93)			
	Mean (SD)	Mean (SD)	*t*	*p*	Hedges’ g	Mean (SD)	Mean (SD)	*t*	*p*	Hedges’ g
Age, y	14.21 (1.41)	14.40 (1.24)	−0.551	0.584	0.142	14.80 (1.30)	14.33 (1.24)	0.774	0.445	0.255
Education, y	8.63 (1.47)	8.91 (1.38)	−0.771	0.444	0.198	9.20 (2.05)	8.87 (1.28)	0.352	0.740	0.116
Dose of antidepressants^1^, mg/d	NA	40.16 (12.99)	NA	NA	NA	42.72 (9.88)	39.74 (13.53)	0.471	0.641	0.156
Pre-treatment BDI score	5.00 (4.51)	36.03 (10.97)	−14.991	<0.001	3.852	29.20 (10.50)	37.17 (10.79)	−1.534	0.135	0.507
Post-treatment BDI score	NA	29.23 (12.17)	NA	NA	NA	11.80 (7.19)	32.13 (10.26)	−4.235	<0.001	1.198

1 Dose of antidepressants were transferred to equivalent fluoxetine dosage.

HCs, healthy controls; MDD, major depressive disorder; BDI, Beck Depression Inventory.

### 3.2 Altered dFC patterns in MDD group

**Figure 1:**
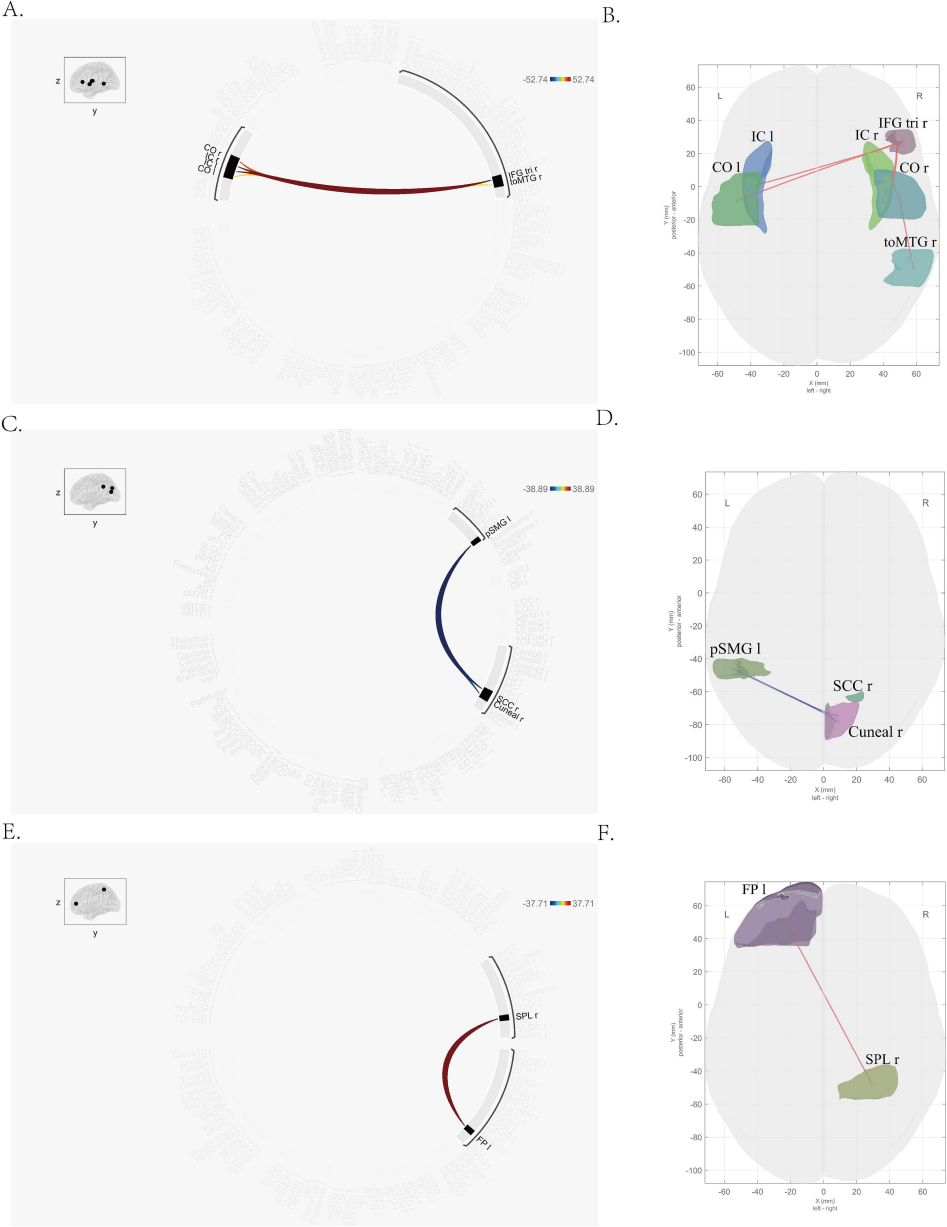
Brain regions with altered dFC pattern after treatment. **(A,B)** Right triangular part of inferior frontal gyrus, bilateral insular cortex, bilateral central opercular cortex, right temporooccipital part of middle temporal gyrus; **(C,D)** right supracalcarine cortex, left posterior division of supramarginal gyrus, right cuneal cortex; **(E,F)** left frontal pole, right superior parietal lobule. The color in picture **A**, **C** and **E** means the mean strength of dFC. Blue stands for the lower dFC, while the red means higher dFC. Significant differences between pre-and post-treatment are shown (two-tailed t-test, *p*-FDR < 0.05).

In this study, seven brain regions (including right temporal occipital fusiform cortex, bilateral planum polar, bilateral planum temporal and bilateral Heschl’s gyrus) with different dFC patterns between MDD and HC group were found, which were listed in Supplementary Table S1. And several brain regions with different dFC patterns in MDD group between pre-and post-treatment were found (Table 2). These brain regions included right triangular part of inferior frontal gyrus (IFG tri r), bilateral insular cortex (right, IC r; left, IC l), bilateral central opercular cortex (right, CO r; left, CO l), right temporooccipital part of middle temporal gyrus (toMTG r), right supracalcarine cortex (SCC r), left posterior division of supramarginal gyrus (pSMG l), right cuneal cortex (Cuneal r), left frontal pole (FP l), right superior parietal lobule (SPL r; Figure 1).

**Table 2:** Connections with different dFC patterns between pre-and post-treatment in MDD group.

Connection	T	*p*
IFG tri r-IC r	4.28	<0.001
IFG tri r-CO r	4.00	<0.001
IFG tri r-IC l	3.42	0.002
IFG tri r-CO l	2.82	0.008
toMTG r– CO r	2.16	0.040
SCC r-pSMG l	−4.28	<0.001
Cuneal r– pSMG l	−3.72	<0.001
FP l-SPL r	4.28	<0.001

IFG tri r, right triangular part of inferior frontal gyrus; IC r, right insular cortex; IC l, left insular cortex; CO r, right central opercular cortex; CO l, left central opercular cortex; toMTG r, right temporooccipital part of middle temporal gyrus; SCC r, right supracalcarine cortex; pSMG l, left posterior division of supramarginal gyrus; Cuneal r, right cuneal cortex; FP l, left frontal pole; SPL r, right superior parietal lobule.

### 3.3 DFC value correlated with BDI score reduction in MDD group

**Figure 2:**
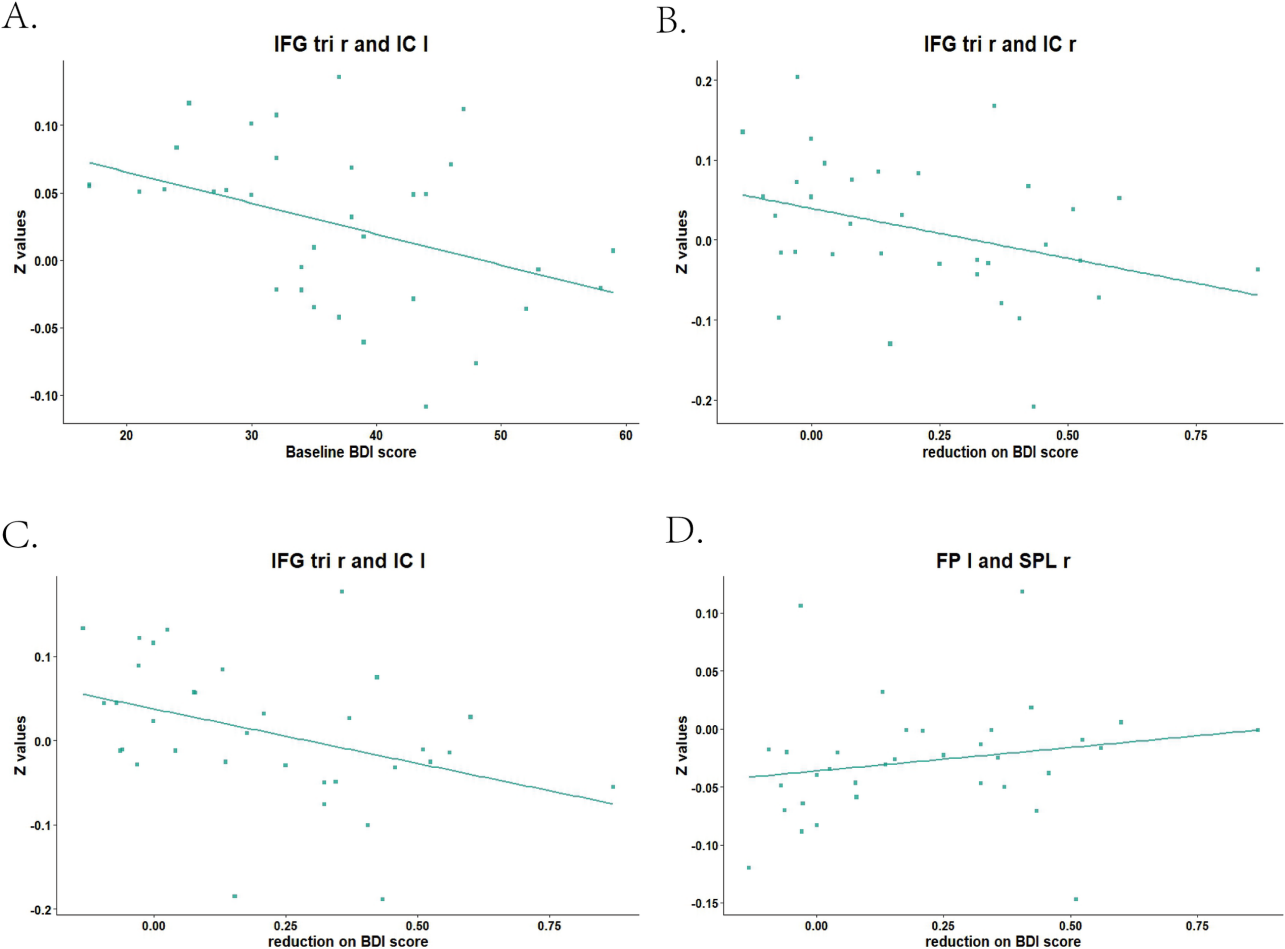
Baseline and reduced BDI score correlated with mean dFC values between brain areas in MDD patients. IFG tri r, right triangular part of inferior frontal gyrus; IC l, left insular cortex; IC r, right insular cortex; FP l, left frontal pole; SPL r, right superior parietal lobule. **(A)** Baseline BDI score correlated with mean dFC value between IFG tri r and IC l; **(B)** Reduction on BDI score correlated with mean dFC value between IFG tri r and IC r; **(C)** Reduction on BDI score correlated with mean dFC value between IFG tri r and IC l; **(D)** Reduction on BDI score correlated with mean dFC value between FP l and SPL r.

Before treatment, we found the mean dFC value between IFG tri r and IC l (*r* = −0.419, *p*-FDR = 0.022) was negatively correlated with BDI score in MDD group (Figure 2A). There were no correlations between mean dFC values and BDI score after treatment.

The mean dFC value between IFG tri r and IC r (*r* = −0.461, *p*-FDR = 0.012) at baseline, as well as which between IFG tri r and IC l (*r* = −0.518, *p*-FDR = 0.007), were negatively correlated with BDI score reduction (Figures 2B,C). The mean dFC value between FP l and SPL r after treatment was positively correlated with BDI score reduction (*r* = 0.442, *p*-FDR = 0.014; Figure 2D).

### 3.4 Prediction of treatment response

#### 3.4.1 Multivariate linear regression analyses

Linear regression models incorporated mean dFC value at baseline as independent variable, BDI score reduction (referred as treatment response) as dependent variable. Covariates included age, sex, baseline BDI score and medication dosage of patients. We found lower mean dFC value between IFG tri r and IC r (Model A; *β* = −1.563, SD = 0.599, *t* = −2.607, *p*-FDR = 0.021, adjusted R^2^ = 0.214) at baseline, as well as which between IFG tri r and IC l (Model B; *β* = −1.868, SD = 0.598, *t* = −3.123, *p*-FDR = 0.012, adjusted R^2^ = 0.279), could predict better antidepressant treatment response.

#### 3.4.2 Repeated 3-fold cross-validation

The correlation between predicted and actual BDI score reduction for Model A was 0.44, for Model B was 0.48.

#### 3.4.3 Permutation test

Permutation test was carried out and similar results were obtained. Mean dFC value between IFG tri r and IC r (*p* = 0.017, *p*-FDR = 0.021) at baseline, as well as which between IFG tri r and IC l (*p* = 0.003, *p*-FDR = 0.012), could predict antidepressant treatment response.

#### 3.4.4 Sensitivity and specificity with BDI score reduction ≥25% as outcome

The sensitivity and specificity for mean dFC value between IFG tri r and IC r were 0.63 and 0.67 respectively, for mean dFC value between IFG tri r and IC l were 0.65 and 0.73, respectively.

## 4 Discussion

In this study, we used dyn-ICA to identify altered dFC patterns (right IFG, bilateral IC, bilateral CO, right MTG, right SCC, left SMG, right cuneal cortex, left FP and right SPL) between pre-and post-treatment in adolescents with MDD. Mean dFC values between right IFG and bilateral IC at baseline was found to be related with antidepressant treatment response and could serve as predictors.

The IFG, IC, and CO are part of the inhibitory control network (ICN; Goghari and MacDonald 3rd, 2009), which is linked to task performance that demand strong inhibitory skills (Congdon et al., 2010). Using the stop-signal task-related probabilistic ICA, Congdon et al. reported two components that included similar regions found in our study (Congdon et al., 2010). They found that activating the ICN was associated with better inhibitory response, the ability to suppress behaviors like motor actions and higher-level responses (such as thoughts and emotions; Congdon et al., 2010). We found dFC between IFG and insular lobe was related with antidepressant treatment. And the right IFG seemed to be a core brain region related to antidepressant treatment. The IFG has a number of functions including the processing of speech and language in Broca’s area (Greenlee et al., 2007). While the insulae is believed to be involved in consciousness and plays a role in diverse functions usually linked to emotion or the regulation of the body’s homeostasis (Bushara et al., 2001). These functions include compassion, empathy, taste, perception, motor control, self-awareness, cognitive functioning, interpersonal relationships, and awareness of homeostatic emotions such as hunger, pain and fatigue (Bushara et al., 2003; De Martino et al., 2006; Xue et al., 2010). Both IFG and insular cortex are well known to be closely related MDD psychopathology (Greenlee et al., 2007; Xue et al., 2010).

The SCC, SMG and cuneal cortex belong to the sensory-motor network (SMN; Chenji et al., 2016). This network includes subnetworks that support sensorimotor abilities, such as the auditory and visual subnetworks (Supekar et al., 2019). The SMN can help individuals identify external stimuli and influence cognitive development in early life (von Hofsten and Rosander, 2018). In patients with MDD, severity of depression symptoms is correlated with connectivity between the executive control network and the SMN (Tang et al., 2021). Moreover, sFC patterns in the SMN were significantly changed in patients with remitted late-life depression and amnestic mild cognitive impairment (Chen et al., 2016; Chen et al., 2020). And these changes were associated with a decline in patient’s overall cognitive functioning and behavioral executive abilities (Chen et al., 2016). Therefore, we speculated that antidepressant might be able to alter SMN activity of affecting patients’ cognitive function, thereby improve their depression symptoms.

Both the FP and SPL are components of the frontoparietal network (FPN), which plays a vital role on targeting in a complex visual environment and is closely associated with cognitive function (Szczepanski et al., 2013). Prior studies have reported that depressed patients have lower connectivity within the FPN compared to HCs (Kaiser et al., 2015b). Moreover, after light therapy, MDD patients showed increased connectivity within the FPN (Huang et al., 2022). In patients with insomnia, connectivity between FP and SPL predicts better treatment response and is positively associated with the score of Pittsburgh Sleep Quality Score (Zheng et al., 2023). And insomnia has been shown to be very closely related to MDD and antidepressants may change FC in FPN to alleviate sleep disturbance in MDD patients (Riemann et al., 2020).

There are several limitations in our study. First, the sample size was relatively small. And the number of responders was consequently low, so we chose 25% as a cut-off value. In future, we will expand sample size to do further exploration. Second, our study only focused on acute treatment response which may contribute to low number of responders. We chose 6 weeks of treatment as the intervention because 6 weeks is a recommended time to change medication, while the original drug is useless (Ogle and Akkerman, 2013). Future research could look at long-term treatment response. Last, our study did not consider the effect of environmental factors. As is suggested in previous study, the onset of depression is influenced by both genes and the environment, including childhood stress, early trauma, shift work and so on (Kaufman et al., 2018; Mitra et al., 2018; Diez et al., 2021). So environmental factors are crucial, especially for children and adolescents. Therefore, future studies should also investigate the effects of environmental factors on treatment response in adolescent MDD.

## 5 Conclusion

In this study, we identified specific dFC pattern-based predictors of pharmacological treatment response involving brain regions known to be affected by MDD. Mean dFC values between right IFG and bilateral IC at baseline were found to be related with antidepressant treatment response and could serve as predictors. And the specific mechanisms underlying the relationship between dFC patterns and treatment response need to be further investigated in the future.

## Data Availability

The raw data supporting the conclusions of this article will be made available by the authors, without undue reservation.

## Footnotes

^1^ http://www.mccauslandcenter.sc.edu/mricro/mricron

^2^ https://www.fil.ion.ucl.ac.uk/spm/software/spm12/

^3^ www.nitrc.org/projects/conn

^4^ https://www.r-project.org/
