# Supplementary material for: Dynamic functional connectivity patterns predict early antidepressant treatment response in drug-naïve, first-episode adolescent MDD
Source: Front Neurosci. 2025 Feb 3;19:1487754. doi: 10.3389/fnins.2025.1487754 (PMC11830731; doi:10.3389/fnins.2025.1487754)
Supplement: Supplementary file 1 [file Table_1.DOCX]

Supplementary Material

**Table S1.** Brain regions with different dFC patterns between MDD and HC group.

| Connection | T | *p* |
| --- | --- | --- |
| TOFusC r– PP r | -3.26 | 0.002 |
| TOFusC r– PT r | -3.06 | 0.003 |
| TOFusC r– HG r | -2.89 | 0.005 |
| TOFusC r– HG l | -2.41 | 0.019 |
| TOFusC r– PT l | -2.18 | 0.033 |
| TOFusC r– PP l | -2.04 | 0.047 |

Abbreviations: TOFusC r, right temporal occipital fusiform cortex; PP r, right planum polar; PT r, right planum temporal; HG r, right Heschl's gyrus; HG l, left Heschl's gyrus; PT l, left planum temporal; PP l, left planum polar.
